# Photonic topological Lifshitz interfaces

**DOI:** 10.1515/nanoph-2021-0807

**Published:** 2022-02-04

**Authors:** Xianji Piao, Jonghwa Shin, Namkyoo Park

**Affiliations:** Photonic Systems Laboratory, Department of Electrical and Computer Engineering, Seoul National University, Seoul 08826, Korea; Department of Materials Science and Engineering, Korea Advanced Institute of Science and Technology, Daejeon 34141, Korea

**Keywords:** Abraham–Minkowski controversy, interface states, Lifshitz transition, topology, transverse spin, wavevector diagram

## Abstract

The intrinsic geometry of wavevector diagrams describes electronic or photonic transport at a given energy level. Lifshitz transition is an intriguing example of the topological transition in wavevector diagrams, which plays a critical role in abnormal transport with enhanced magnetoresistance or superconductivity. Here, we develop the spatial analogy of the Lifshitz transition, which provides a comprehensive topological perspective on transverse-spin interface states. We establish the excitation conditions of transverse-spin interface states, which require the “Lifshitz interface” – the interface between different topologies of wavevector diagrams – along with the gap in wavevector diagrams. Based on the detailed analysis of this topological phenomenon with respect to the dimensionality and gaps of wavevector diagrams across the Lifshitz interface, we show distinct parity of transverse spins and power flows in transverse-spin modes. The unique symmetry of interface states realizing Abraham-spin-momentum locking represents the gauge induced by the Lifshitz interface, which provides a novel insight into the Abraham–Minkowski controversy.

## 1 Introduction

Transport properties of electrons or photons at a given energy level are described by wavevector diagrams: an electronic Fermi surface or a photonic isofrequency surface (IFS), both of which represent the density of states in reciprocal spaces. The intrinsic geometries of these surfaces [1] are determined by material or structural parameters, such as lattice deformations or pressure-dependent anisotropy [2], anisotropic material tensors [3], and crystalline structures [4]. Various intrinsic geometries of wavevector diagrams are obtained even with homogeneous media, including an ellipsoid with a closed topology, a hyperboloid with an open topology, and a singular point with zero dimensionality. Such topologies of wavevector diagrams govern wave behaviors, stimulating subwavelength confinements in hyperbolic metamaterials [5] and anomalous diffractions in photonic crystals [6], [7], [8]. The topological transition between wavevector diagrams having different intrinsic geometries has especially attracted significant attention. In condensed-matter physics, the Lifshitz transition [2] – the sudden change between open and closed topologies of Fermi surfaces – leads to transport anomalies in superconductors [9] and semimetals [10]. This notion has been generalized to the dynamical transitions between Fermi arcs, Weyl points, and Dirac points of different topologies [11], [12], [13], [14].

In photonics, the analogy of the Lifshitz transition has been applied to control light transport [5, 15], [16], [17]. Various spatial transitions of the IFSs have been studied to manipulate Poynting vectors [18], transverse spin (T-spin) angular momenta [19, 20], and modal properties of surface plasmons or Dyakonov states [21, 22]. The excitation of a spatially pure T-spin wave was also demonstrated with the inversely-designed platform [23]. However, previous approaches have focused on specific configurations, lacking the inspiration from the notions of the Lifshitz transition and its topology. Considering a fertile ground of topological photonics [24], [25], [26] developed from the interplay between two critical natures of dispersion bands – topology and gap – a viewpoint stemming from topological photonics will provide a deeper understanding of spatial Lifshitz transition. At the same time, when considering the universal definition of topology – geometric properties preserved under continuous deformation – the analogy of topological wave phenomena in terms of the Lifshitz transition will stimulate the extension of topological photonics into various classes of topologies, not restricted to the topology of the IFS [5, 27]. Notably, although there have been efforts to examine conventional topological quantities (i.e., Chern number) in the momentum space [28], the approach of utilizing the topology of the wavevector diagrams, focusing on extending topological photonics, is yet to be explored.

In this paper, we establish the notion of “Lifshitz interfaces” – a class of spatial boundaries between different IFS topologies – which covers the environments for surface-plasmon [29], Dyakonov [21, 22], and pure T-spin waves [23]. As an analogy of topologically-protected interface states in topological bandgap materials [24, 25, 30], we show that the Lifshitz interface with overlapped gaps between wavevector diagrams leads to a T-spin interface mode. We also reveal the critical role of the IFS dimensionality on handling this topological phenomenon, which differentiates the parity of power flows and T-spin modes while preserving spin-momentum locking. Our result provides an intuition on the role of the IFS topologies in elucidating the discrepancy between the Abraham and Minkowski interpretations of optical momenta.

## 2 Results

### 2.1 Topology and gap of wavevector diagrams

**Figure 1:**
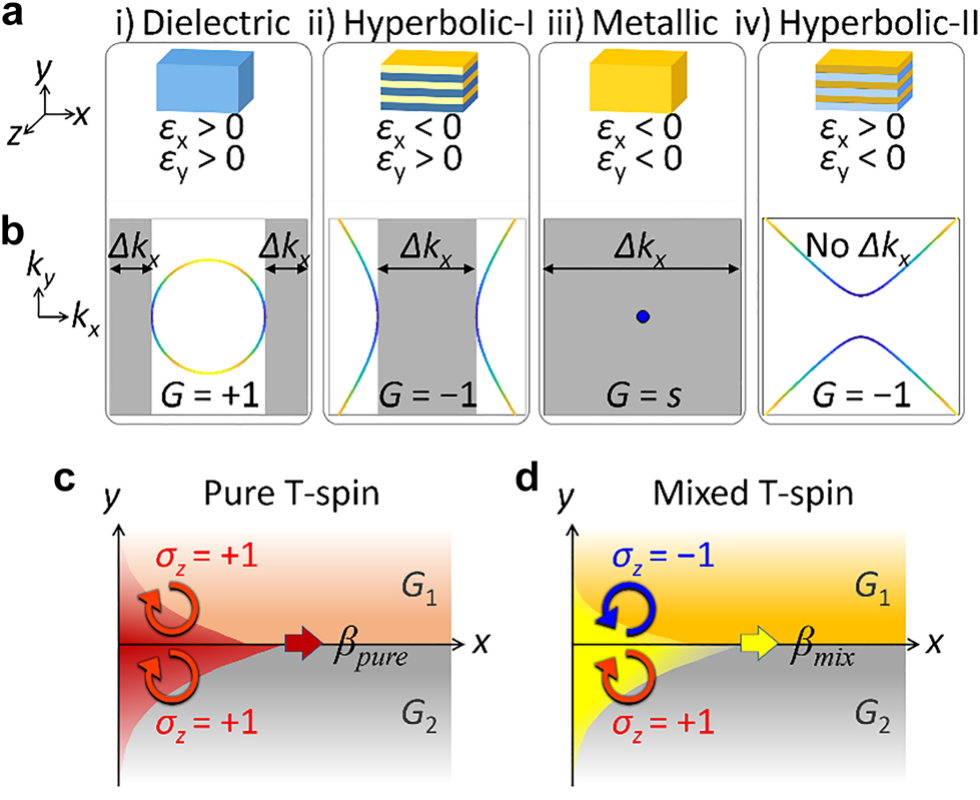
Topology and gap of IFCs. (a) Anisotropic media: dielectric (*ε*_*x*_ > 0, *ε*_*y*_ > 0), hyperbolic I (*ε*_*x*_ < 0, *ε*_*y*_ > 0), metallic (*ε*_*x*_ < 0, *ε*_*y*_ < 0), and hyperbolic II (*ε*_*x*_ > 0, *ε*_*y*_ < 0) media. (b) IFC diagrams and their wavevector gaps (Δ*k*_*x*_, gray regions). (c and d) Two T-spin interface states: (c) a pure T-spin mode and (d) a mixed T-spin mode. Blue and red arrows denote sgn(*σ*_*z*_) = +1 and sgn(*σ*_*z*_) = −1 spin handedness, respectively, where *σ*_*z*_ denotes the handedness of T-spin modes.

We consider a two-dimensional (2D) homogeneous nonmagnetic medium with anisotropic permittivities *ε*_*x*,*y*_. This material allows four different types of isofrequency contours (IFCs), the 2D projection of IFSs, having different topologies of wavevector diagrams (Figure 1a and b): (i) a closed topology of an elliptical IFC with dielectric media, (ii, iv) two open topologies of hyperbolic IFCs (type I and II), and (iii) a singular point with a metallic medium. To identify the topology of each IFC, we use the Gaussian curvature *Κ* [31] (see Supplement 1, Section A.1). While *Κ* measures the deviation of a surface from a plane (*Κ* > 0 for an elliptical surface and *Κ* < 0 for a hyperbolic surface), we utilize a geometrical indicator *G* = *Κ*/|*Κ*| (Figure 1b; *G* = 1 for elliptic media, *G* = −1 for hyperbolic media, and *G* = *s* (*singular*) for metallic media). Notably, the continuous deformation of each geometry does not allow the transition to the geometry with the other *G*, exhibiting the topological nature of IFCs [32]. As widely studied in quantum-optical analogy [33], we develop the spatial analogy of the dynamical Lifshitz transition, by constructing the abrupt topological transition of the IFC: “Lifshitz interface”. This realization not only provides the spatial photonic analogy of the Lifshitz transition but also extends the concept of a topological interface, which conventionally describes the interface between materials with different knotted properties of wavefunctions in dispersion bands [24].

In the analogy of topological photonics, a critical factor is the existence of “gaps”, as shown in topologically-protected states at the boundary between bandgap materials [24]. While the class of the IFC topology is fundamentally different from dispersion band topologies, we can introduce the direct analogy of bandgaps by employing forbidden ranges in wavevector spaces. Analogous to the energy gap of a *k*–*ω* dispersion that depicts the forbidden *ω* range, each IFC at a constant *ω* can have forbidden *k* ranges, realizing a “wavevector gap” Δ*k*_*x*_ (gray region in Figure 1b) for *x*-axis propagations. As similar to topologically-protected interface states at the topological boundary between bandgap materials, we envisage the emergence of interface states in wavevector gaps involved with the nontrivial topological contact of IFC geometries. Figure 1c and d show two interface states obtained from the proposed configuration, exhibiting distinct parity for T-spin handedness. Although these states have been studied separately in terms of the T-spin states [23] and surface-plasmon-like modes [29], their interpretation in terms of topological Lifshitz interfaces is absent.

### 2.2 Lifshitz interface at the same IFC dimensionality

**Figure 2:**
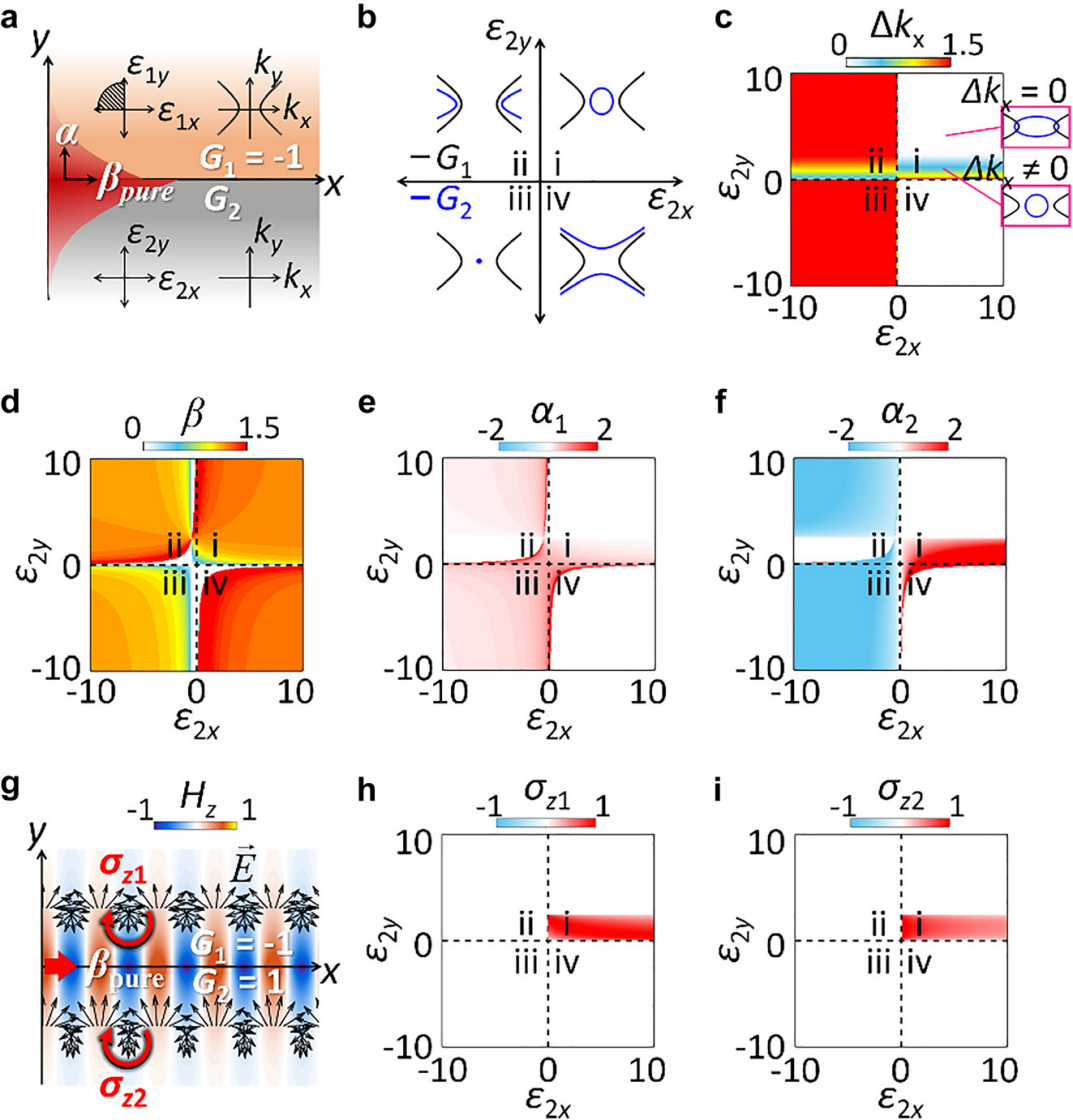
Lifshitz interface at the same IFC dimensionality. (a) An interface between a hyperbolic-I layer (*G*_1_ = −1) and a varying layer (*G*_2_). (b) Four quadrants of IFCs in the material parameter space (i: *G*_2_ = 1, ii: *G*_2_ = −1, iii: *G*_2_ = *s*, iv: *G*_2_ = −1) with the hyperbolic-I top layer (*G*_1_ = −1). (c–f) Phase diagrams for (c) wavevector gap Δ*k*_*x*_, (d) propagation constant *β*, and (e and f) decay factors of (e) *α*_1_ and (f) *α*_2_. (g) Magnetic field profile *H*_*z*_ for a pure T-spin mode at the Lifshitz interface having the same IFC dimensionality (*G*_1_ = −1 with *ε*_1*x*_ = −0.5, *ε*_1*y*_ = 2.5, and *G*_2_ = 1 with *ε*_2*x*_ = 0.5 *ε*_2*y*_ = 1.5). Black and red arrows denote *E*-field and the T-spin handedness, respectively. (h and i) Phase diagrams for T-spin density *σ*_*z*1_ and *σ*_*z*2_. In (c–f, h, and i), the top layer has *ε*_1*x*_ = −0.5 and *ε*_1*y*_ = 2.5.

Between the two interface states with distinct parity of T-spin handedness (Figure 1c and d), we first investigate the pure spin mode (*G*_1_ = −1, *G*_2_ = 1, Figure 1c) achieved with the interface between the materials, both having 2D IFCs. Although an accidental excitation of the pure T-spin mode was reported using the inverse design technique [23], the work lacks the general criteria of the excitation condition. To generalize the result in [23] to the notion of the Lifshitz interface, we explore the following configuration: the interface between a hyperbolic-I top layer (*G*_1_ = −1) and a varying bottom layer (*G*_2_) (Figure 2a). Among four possible types of interfaces depending on the IFC topologies of the bottom layer (Figure 2b), the first (*G*_1_ = −1, *G*_2_ = 1) and the third (*G*_1_ = −1, *G*_2_ = *s*) quadrants compose the Lifshitz interfaces, while the former one has the IFC geometry at the same dimensionality. The pure T-spin state occurs at the interfaces of (*G*_1_ = −1, *G*_2_ = 1), which represents the spatial topological transition between hyperbolic and elliptical IFCs (Figure 2b).

To establish the excitation conditions of pure T-spin modes, we examine the phase diagrams defined in the material-parameter space (Figure 2c–f): wavevector gap (Δ*k*_*x*_), propagation constant (*β* = *k*_*x*_/*k*_0_), and decay factors (*α*_1_ = *k*_*y*1_/*k*_0_, *α*_2_ = −*k*_*y*2_/*k*_0_), where *k*_*x*_ and *k*_*y*1,2_ denote the *x*-axis wavevector and *y*-axis decay constants to the layer 1 and 2, respectively (see Supplement 1, Section B.1 and Section B.2). These phase diagrams provide the excitation criteria of T-spin modes in terms of the analogy of topological photonics: the interplay between IFC topologies and wavevector gaps.

The wavevector gap Δ*k*_*x*_ between different IFC geometries is determined with material parameters, e.g., *ε*_2*x*_ and *ε*_2*y*_ (Figure 2c, see Supplement 1, Section A.2), showing gaps in a part of the first quadrant of *ε*_2*x*_–*ε*_2*y*_ (i region) and the entire region of the second and third quadrants of *ε*_2*x*_–*ε*_2*y*_ (ii and iii regions). Among these gap regimes, the *x*-axis propagating bound modes (*β* > 0 in Figure 2d and *α*_1,2_ > 0 in Figure 2e and f) are achieved only in the part of the first quadrant, which exclusively satisfies the interplay between a Lifshitz interface (*G*_1_ ≠ *G*_2_) and a wavevector gap (Δ*k*_*x*_ ≠ 0). This condition represents an intriguing analogy of conventional topological photonics: interface states from the interplay between the nontrivial interface of band topologies and the emergence of bandgaps [24].

We note that the observed states at the Lifshitz interfaces with the same IFC dimensionality possess spatially “pure” T-spin across the interface (Figure 2g): even-parity symmetry sgn(*σ*_*z*1,*z*2_) = +1, where *σ*_*z*1,2_ are the local spin density for an electric field in the layer 1 and 2, respectively (Figure 2h and i, see Supplement 1, Section B.3). Due to the robustness of the topological states, the T-spin excitation is protected within the entire regime of the Lifshitz interface with wavevector gaps (Figure 2h and i), also leading to the spin-momentum locking (here, *σ*_*z*_ > 0 for *β* > 0). As demonstrated in [29, 34], the observed spin-momentum locking also enables the unidirectional excitation of interface states by changing the incidence angle or light polarization.

### 2.3 Lifshitz interface at different dimensionalities

**Figure 3:**
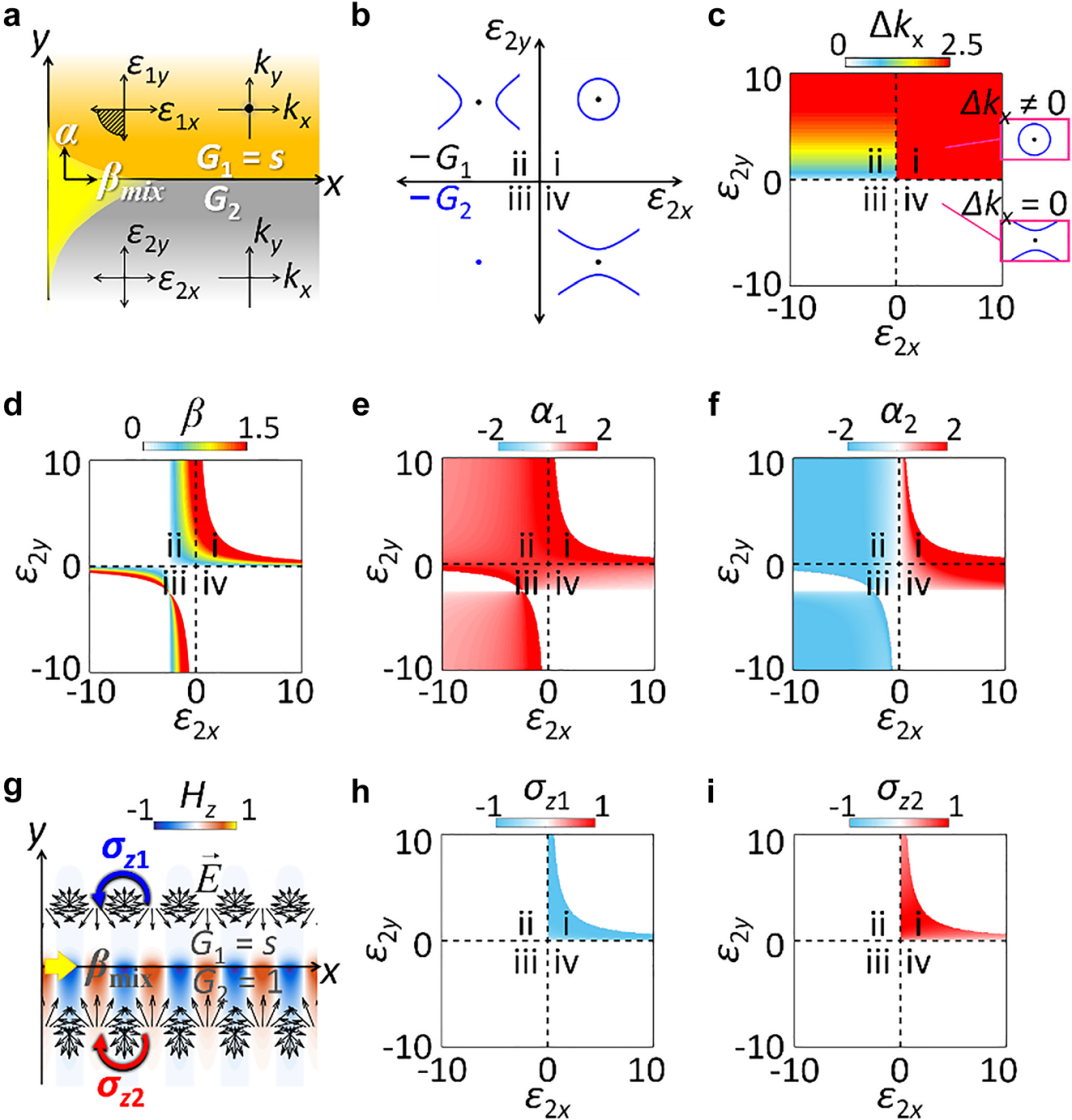
Lifshitz interface at different IFC dimensionalities. (a) An interface between a metallic layer (*G*_1_ = *s*) and a varying layer (*G*_2_). (b) Four quadrants of IFCs in the material parameter space of the varying bottom layer (i: *G*_2_ = 1, ii: *G*_2_ = −1, iii: *G*_2_ = *s*, iv: *G*_2_ = −1), with the metal top layer (*G*_1_ = *s*). (c–f) Phase diagrams for (c) Δ*k*_*x*_, (d) *β*, (e) *α*_1_, and (f) *α*_2_. (g) *H*_*z*_ profile for a mixed T-spin mode at the Lifshitz interface having different IFC dimensionalities (*G*_1_ = *s* with *ε*_1*x*_ = −2, *ε*_1*y*_ = −2, and *G*_2_ = 1 with *ε*_2*x*_ = 0.5 *ε*_2*y*_ = 1.5). Black and blue/red arrows denote *E*-field and the T-spin handedness. (h and i) Phase diagrams for T-spin density *σ*_*z*1_ and *σ*_*z*2_. In (c–f, h, and i), the top layer has *ε*_1*x*_ = −2 and *ε*_1*y*_ = −2.

The Lifshitz interfaces at different IFC dimensionalities lead to a mixed T-spin mode (Figure 1d), which includes the familiar surface plasmon at a metal–dielectric interface (*G*_1_ = *s*, *G*_2_ = 1) [35]. In terms of the Lifshitz interface, we investigate a class of interfaces between a metal (*G*_1_ = *s*) and a varying material (*G*_2_) (Figure 3a). Among the four types of interfaces depending on the varying *G*_2_ (Figure 3b), three of them (i, ii, and iv regions) are associated with the topological transitions between different dimensional IFCs.

We again investigate the phase diagrams defined in the material-parameter space (Figure 3c–f). Because the IFC geometry of metals is a point, the wavevector gap of the interface is solely determined by the IFC of the bottom layer (Figure 3c), leading to Δ*k*_*x*_ in the first and second quadrants. As identical to the discussion in Figure 2, the excitation of the interface states follows the principle of topological photonics; *x*-axis propagating interface states (*β* > 0 in Figure 3d and *α*_1,2_ > 0 in Figure 3e and f) are achieved only in the part of the first quadrant, satisfying the interplay between a Lifshitz interface (*G*_1_ ≠ *G*_2_) and a wavevector gap (Δ*k*_*x*_ ≠ 0).

While the interface state in Figure 3 is also topologically protected same as Figure 2, the difference in the dimensionality across the Lifshitz interface imposes the uniqueness on the T-spin mode of the observed state (Figure 3g–i). In contrast to the pure T-spin mode, the contact of metal (*G*_1_ = *s*) and dielectric (*G*_2_ = 1) layers results in the mixed T-spin mode having odd parity in the spin handedness (sgn(*σ*_*z*1_) = −1 and sgn(*σ*_*z*2_) = 1). This odd parity of the T-spin mode originates from the transition between different dimensional topologies. In contrast to ℤ_2_ insulators, which treat topological invariants in the same dimensionality, the Lifshitz interface involves the topologies at different dimensionalities, such as 2D Fermi surfaces, 1D Fermi arcs, and 0D Weyl and Dirac points [5, 12, 14]. This result inspires the generalization of topological interfaces by including the contribution of dimensionalities.

### 2.4 Abraham–Minkowski controversy in T-spin modes

**Figure 4:**
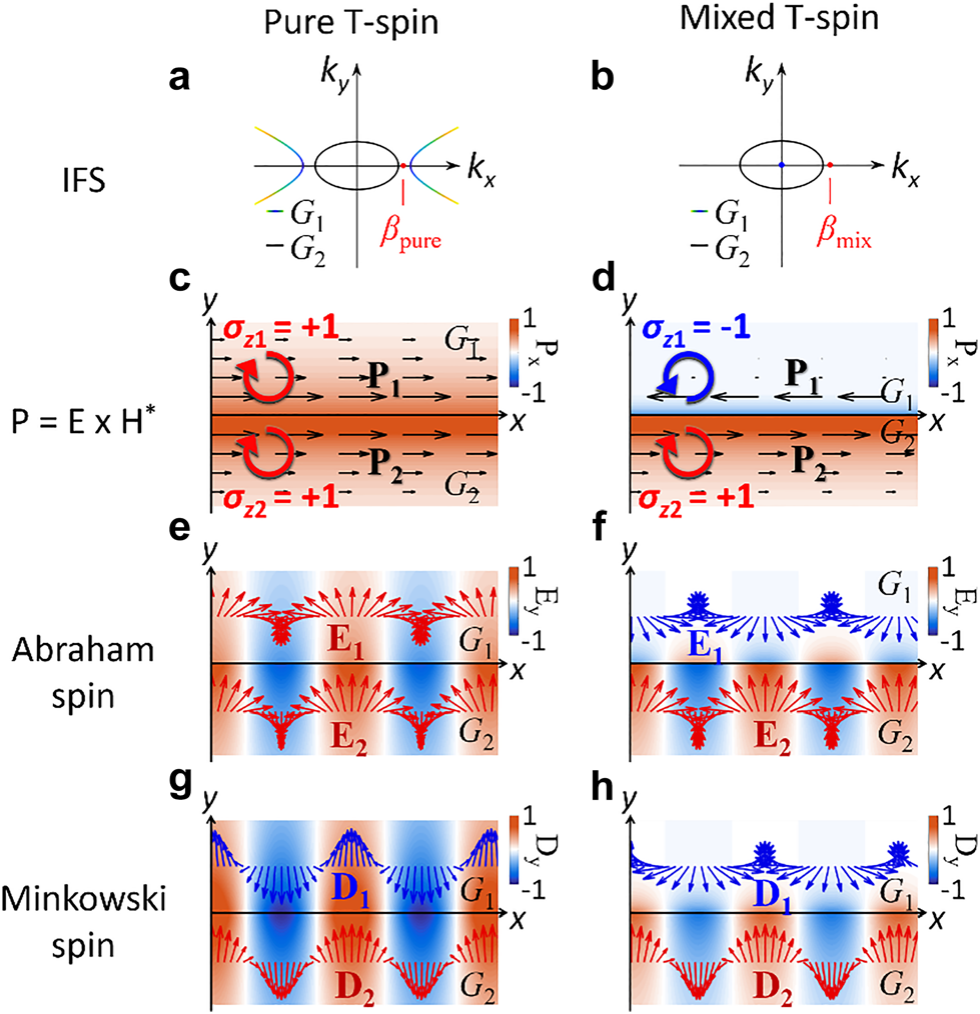
Abraham–Minkowski discrepancy in the Lifshitz interfaces. (a and b) Wavevector diagrams of interfaces with (a) pure T-spin modes and (b) mixed T-spin modes. The red dots represent the interface states. Distributions of (c and d) *P*_*x*_ = **P**∙**e**_*x*_, (e and f) Re(*E*_*y*_), and (g and h) Re(*D*_*y*_) for two types of T-spin modes: (c, e, and g) a pure T-spin mode at a hyperbolic–elliptic interface and (d, f, and h) a mixed spin mode at a metal–elliptic interface. In (c and d), the black arrows depict the Poynting vectors **P**_**1,2**_, on each side of the interface. In (e–h), blue and red arrows on each side of the interface represent different spin handedness of **E** and **D**.

In terms of the analogy of topological photonics with the Lifshitz interface platforms, the results in Section 2.1–2.3 demonstrate the interplay between topologically nontrivial interfaces defined by the IFC geometries and their wavevector gaps. Especially, the uniqueness of the topological Lifshitz interface states in relation to the IFC dimensionality is also clarified with their power flows and spin angular momenta. We note that a hyperbolic–elliptic interface (Figure 4a) and a metallic–elliptic interface (Figure 4b) for IFC-topological Δ*k*_*x*_-gap materials lead to distinct distributions of power flows. While a pure T-spin mode has an even parity for Poynting vectors **P** (Figure 4c), a mixed T-spin mode has an odd parity (Figure 4d). The electric-field-spinning quantified by *σ*_*z*_ demonstrates spin-momentum locking [35], [36], [37], [38] for all cases (sgn(*σ*_*z*_)sgn(**P**∙**e**_*x*_) > 0 for all *y*).

However, because optical momenta have been traditionally interpreted in two ways – Abraham [39] and Minkowski [40] interpretations – it is necessary to examine the resulting spin states with both definitions. Notably, two types of T-spin modes provide an intriguing discrepancy in interpreting optical spin momenta inside a material, which has stimulated a longstanding controversy between the Abraham [39] and Minkowski [40] interpretations. While the Minkowski view considers a momentum representation in the form **D** × **B** to express the “wave momentum” inside a material [40], the Abraham view relies on the **E** × **H** representation as the “true momentum” of light [41].

For a transverse magnetic mode in a nonmagnetic medium (**B** = *μ*_0_ **H**), the spin angular momentum in each interpretation is determined by the spinning of the electric field **E** or displacement field **D**. By far, our expression of the T-spin mode follow the Abraham’s view (Figure 4e and f), satisfying spin-momentum locking. When compared to the Minkowski forms of two T-spin modes at Lifshitz interfaces, an intriguing Abraham–Minkowski discrepancy is observed, showing different spin mode symmetry between two interpretations in the pure T-spin mode (Figure 4e and g). On the contrary, the mixed T-spin mode shows agreement between the Abraham and Minkowski interpretations, supporting the same spin mode symmetry (Figure 4f and h). The results in Figure 4c–h therefore imply that the Abraham’s view is more proper to interpret the spin-momentum locking originating from the topological Lifshitz interface, because the contributions from material are already included in the IFC defined by the permittivity tensor.

Based on the analysis in Figures 2 and 3, we attribute the origin of such a discrepancy to the dimensionality of IFCs: the hyperbolic–elliptic interface as an iso-dimensional Lifshitz interface and the metallic–elliptic interface as a hetero-dimensional Lifshitz interface. When considering the discrepancy between the Abraham (**p**_**Abraham**_ = **p**_**total**_ − **p**_**kin**_) and Minkowski (**p**_**Minkowski**_ = **p**_**total**_ − **p**_**can**_) definitions [42] – the remaining part of the conserved total light–matter momentum (**p**_**total**_) after the subtraction of either the kinetic (**p**_**kin**_) or canonical (**p**_**can**_) momentum of “matter” – we emphasize that the difference between Abraham and Minkowski representations corresponds to a gauge potential of anisotropic matter that change the ratio between the kinetic and canonical momenta of matter. This gauge field induced by the Lifshitz interface results in the complete analogy of the photonic quantum spin Hall effect and spin-momentum locking in wavevector diagrams.

## 3 Conclusions

We revealed the T-spin-mode arising at the spatial interfaces between topologically distinct IFS materials. Employing the topology of IFSs and the topological transitions between them, we developed the concept of the Lifshitz interface as the spatial-domain realization of the Lifshitz transition. We showed that the interplay between topologically nontrivial interfaces and wavevector gaps leads to T-spin interface wave transport, analogous to ℤ_2_ topological insulators. We also reported the distinct distributions of T-spin mode around the interface with Abraham and Minkowski representations. The results imply the suitability of Abraham momenta for elucidating topological properties of the Lifshitz interfaces, such as spin-momentum locking and the existence of a gauge field to interpret the discrepancy of Abraham–Minkowski representations.

In terms of the complete analogy of topological photonics, further studies on the bulk-edge correspondence will provide a concrete foundation on utilizing the geometric topology of the IFS. In order to demonstrate the bulk-edge correspondence – the difference between forward and backward interface states determined by the topological structure of the bulk states [30] – which requires multiple nontrivial topological invariants, it is necessary to generalize the current platform, for example, by including inhomogeneous and anisotropic materials, such as photonic crystals. Our viewpoint will therefore be extended to more complex IFS geometries involving inhomogeneous media and momentum gaps in time crystals.

## Supplementary Material

Supplementary Material
