# Supplementary material for: Photonic topological Lifshitz interfaces
Source: Nanophotonics. 2022 Feb 4;11(6):1211–7. doi: 10.1515/nanoph-2021-0807 (PMC11501609; doi:10.1515/nanoph-2021-0807)
Supplement: Supplementary file 1 — Supplementary Material [file j_nanoph-2021-0807_suppl.pdf]

Photonic Topological Lifshitz Interfaces: supplemental document

Xianji Piao,[1] Jonghwa Shin,[2] Namkyoo Park[1,*]

[1]*Photonic Systems Laboratory, Dept. of Electrical and Computer Engineering, Seoul National University, Seoul 08826, Korea*

[2]*Department of Materials Science and Engineering, Korea Advanced Institute of Science and Technology, Daejeon 34141, Korea*

**Corresponding author: nkpark@snu.ac.kr*

A. Gaussian curvature and gap dynamics of IFS

A.1. Gaussian curvature of IFS

In differential geometry, the Gaussian curvature of a surface is defined as $K = \kappa_1\kappa_2$, where κ_1 and κ_2 are the principal curvatures at the given point. In the main text, we focus on the IFSs $k_x^2/\varepsilon_y + k_y^2/\varepsilon_x + k_z^2/\varepsilon_z = k_0^2$, especially for uniaxial anisotropic materials, namely $\varepsilon_x \neq \varepsilon_y = \varepsilon_z$. The Gaussian curvature of an elliptical IFS ($\varepsilon_x > 0$, $\varepsilon_y = \varepsilon_z > 0$, Fig. S1a) is

$$K = k_0^2 \frac{\varepsilon_x\varepsilon_y\varepsilon_z}{[(\varepsilon_x\varepsilon_z/\varepsilon_y)k_x^2 + (\varepsilon_y\varepsilon_z/\varepsilon_x)k_y^2 + (\varepsilon_x\varepsilon_y/\varepsilon_z)k_z^2]^2}\,, \tag{S1}$$

while the curvature for a hyperbolic IFS ($\varepsilon_x < 0$ and $\varepsilon_y = \varepsilon_z > 0$, Fig. S1b) is

$$K = \frac{k_0^2}{\varepsilon_x\varepsilon_y\varepsilon_z}\frac{1}{(k_x^2/\varepsilon_y^2 + k_y^2/\varepsilon_x^2 + k_z^2/\varepsilon_z^2)^2}\,. \tag{S2}$$

In the main text, we apply a geometric indicator $G = K/|K|$ to represent the geometrical topology of IFSs.

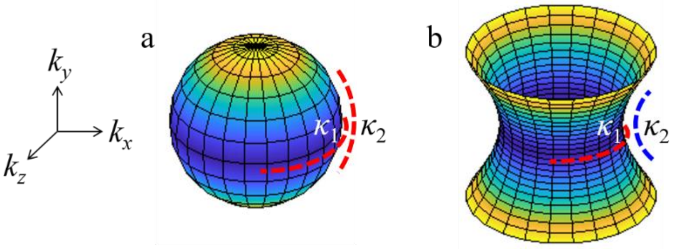

Fig. S1. Schematics of principal curvatures κ_1, κ_2 for (a) elliptical and (b) hyperbolic IFSs.

A.2. Wavevector gap of IFS

In the main text, we introduce the concept of wavevector gap (Δk_x) for the x-propagating interface states in the k_x-k_y two-dimensional (2D) IFC diagrams (Fig. 1b). Analogous to the energy gap in a k-ω dispersion diagram describing the forbidden range of the frequency ω, each IFS at a constant frequency has a forbidden regime of propagating states. The position and magnitude of Δk_x depends on the material constants. For example, the Δk_x of an elliptical IFS spans the outside of the ellipse along the k_x-axis: Δk_x: $\{k_x \mid k_x < -k_e, k_x < k_e\}$, where $k_e = |\varepsilon_y|k_0$ is the magnitude of the vertex of the elliptical IFC in the k_x-axis. The type-I hyperbolic IFS has a single span of Δk_x: $\{(k_x \mid -k_h < k_x < k_h\}$ with $k_h = |\varepsilon_y|k_0$, while no Δk_x for the type-II hyperbolic IFS. For metallic media, the Δk_x spans all the range of k_x in consideration except for the origin point.

For an interface, Δk_x is defined as the overlap of Δk_x in environmental layers. The propagation constant of the pure T-spin state β_{pure} lies inside the gap Δk_x: $\{k_x \mid k_e < k_x < k_h\}$, where $k_h = |\varepsilon_{1y}|k_0$ and $k_e = |\varepsilon_{2y}|k_0$ are the magnitude of the vertices of the hyperbolic-I and the elliptical IFCs, respectively (Fig. S2a). The material constants of this example structure for the pure T-spin states are $\varepsilon_{1x} = -0.5$, $\varepsilon_{1y} = 2.5$, $\varepsilon_{2x} = 0.5$, and $\varepsilon_{2y} = 1.5$. In the metal-dielectric interface, the propagation constant of the mixed T-spin state also lies inside the gap Δk_x: $\{k_x \mid k_x < -k_e, k_x < k_e\}$, where $k_e = |\varepsilon_y|k_0$ (Fig. S2b). We choose an example structure for the excitation of mixed T-spin waves with $\varepsilon_{1x} = -2.0$, $\varepsilon_{1y} = -2.0$, $\varepsilon_{2x} = 0.5$, $\varepsilon_{2y} = 1.5$ (Fig. S2b).

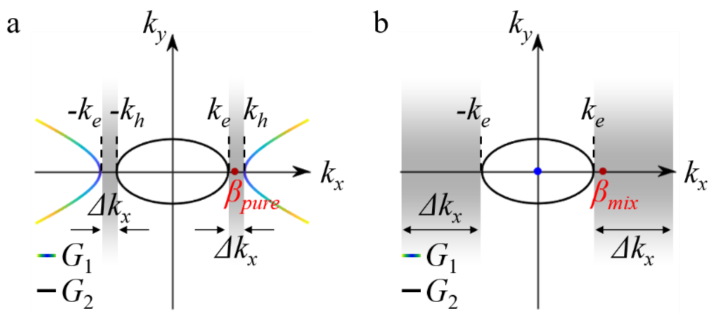

Fig. S2. Wavevector gaps of IFCs for (a) pure T-spin states and (b) mixed T-spin states.

B. Wave quantities for bound interface states

In the main text, we focus on a transverse magnetic (TM) mode that propagates along the interface (x-axis) between two nonmagnetic ($\mu = \mu_0$) anisotropic media with permittivity tensors $\boldsymbol{\varepsilon}_1$ and $\boldsymbol{\varepsilon}_2$. For each medium, we assume uniaxial anisotropic medium for the formulation of the permittivity tensor with $\varepsilon_y = \varepsilon_z$ (Fig. S3).

B.1. Dispersion relation of the x-propagating interface state

The dispersion relation of this TM interface mode is

$$\beta^2 = k_0^2 \frac{\varepsilon_{1y}\varepsilon_{2y}(\varepsilon_{1x} - \varepsilon_{2x})}{\varepsilon_{1x}\varepsilon_{1y} - \varepsilon_{2x}\varepsilon_{2y}}, \tag{S3}$$

where $\varepsilon_{x,y,z}$ is the component of the permittivity tensor and k_0 is the free-space wavenumber.

B.2. Transverse field-decay factors for evanescent interface states

The transverse decay factors of an evanescent wave are $\alpha_1 = [\varepsilon_{1x}(\beta^2/\varepsilon_{1y} - k_0^2)]^{1/2}/k_0$ for $y > 0$, and $\alpha_2 = -[\varepsilon_{2x}(\beta^2/\varepsilon_{2y} - k_0^2)]^{1/2}/k_0$ for $y < 0$. The signs of these decay factors determine the confinement of the excited wave at the interface. When the decay factors have the same sign of values $\mathrm{sgn}(\alpha_1) = \mathrm{sgn}(\alpha_2)$, the excited interface wave decays away from the interface, corresponding to a bound evanescent wave. The other regions with different signs $\mathrm{sgn}(\alpha_1) \neq \mathrm{sgn}(\alpha_2)$ denote unbound modes.

B.3. Spinor representation for T-spin states:

The electrical fields have the form $\boldsymbol{E}(x, y) = \boldsymbol{\Psi}(y)e^{-ikx}$ with the amplitude vector $\boldsymbol{\Psi} = E_+\mathbf{e}_+ + E_-\mathbf{e}_-$, where $\mathbf{e}_\pm = (\mathbf{e}_x \pm i\mathbf{e}_y)$ represent two opposite transverse spinors along the $\pm z$-axis. We note that our representation of the spin component in terms of the electric field follows the Abraham representation of the momentum of light in an anisotropic medium [1, 2]. Because of the duality between electric and magnetic fields, all of the results in our work can be applied to the spinning of a magnetic field by considering uniaxial anisotropic materials with the permeability tensor [3]. The local T-spin density is defined as $\sigma_z = (|E_+|^2 - |E_-|^2) / (|E_+|^2 + |E_-|^2)$ for a $+x$-propagating ($\beta > 0$) interface state, where $E_\pm = E_x \pm iE_y$. The spin density map of σ_{z1} and σ_{z2} in the main text represents the spatial average of the local spin density along y-axis in the layer 1 and 2, respectively.

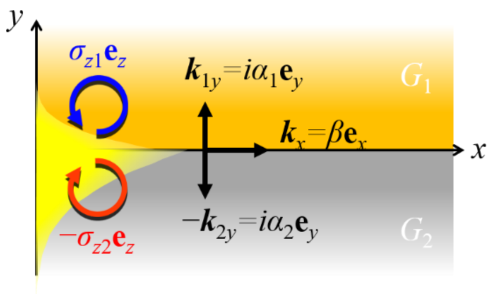

Fig. S3. Wave quantities (β, α_1, and α_2) and spinor representations (σ_{z1}, σ_{z2}) for T-spin interface states.

References

1. S. M. Barnett, "Resolution of the Abraham-Minkowski dilemma," Phys. Rev. Lett. **104**, 070401 (2010).
2. X. Piao, S. Yu, and N. Park, "Design of Transverse Spinning of Light with Globally Unique Handedness," Phys. Rev. Lett. **120**, 203901 (2018).
3. K. Y. Bliokh, A. Y. Bekshaev, and F. Nori, "Dual electromagnetism: helicity, spin, momentum and angular momentum," New Journal of Physics **15**, 033026 (2013).
